# Supplementary material for: Validation of an HPLC Method for the Simultaneous Quantification of Metabolic Reaction Products Catalysed by CYP2C11 Enzymes in Rat Liver Microsomes: In Vitro Inhibitory Effect of Salicylic Acid on CYP2C11 Enzyme
Source: Molecules. 2019 Nov 25;24(23):4294. doi: 10.3390/molecules24234294 (PMC6930622; doi:10.3390/molecules24234294)
Supplement: Supplementary file 1 [file molecules-24-04294-s001.pdf]

Supplementary Materials:

1. UV-VIS Spectroscopy:

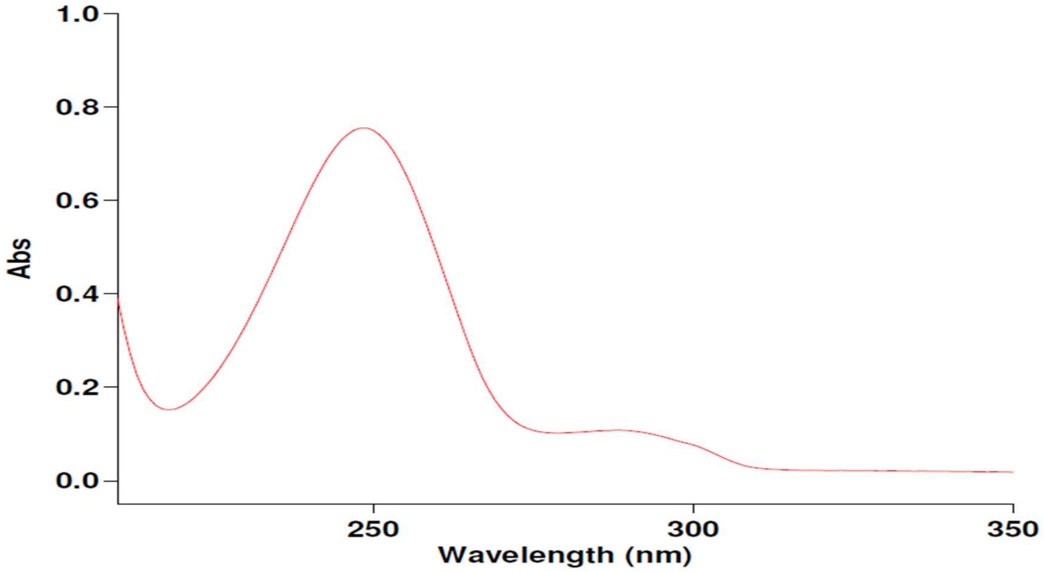

Figure 1. UV-VIS spectroscopy for Phenacetin (internal standard) in the CYP2C11 assay.

2. CYP2C11 control study incubation:

Different concentrations of testosterone (400, 300, 200, 150, 100, 50 and 25µM) were used in CYP2C11 incubation mixture at 60 mins of incubation time using 1.0mM NADPH, 5.0mM G6P, 1.7units/ml G6PDH, 1.0mM EDTA and 3.0mM Magnesium chloride.

Table 1 shows the regression analysis on calibration curves and limit of detection (n=3) for both Testosterone (CYP2C11 substrate) and 16-alfa hydroxytestosterone (CYP2C11 metabolite) for the CYP2C11 assay incubation.

Table 1. Linear regression, range and limit of detection analysis for CYP2C11 assay incubation.

Components	Regression equation (y=ax+b)[a]	R^2	Linear range (µM)	LOD (µM)
Testosterone	y=0.0218x+0.0709	0.9993	25 - 500	8.5972
16α-hydroxytestosterone	y=0.0204x-0.0036	0.9998	10 - 100	1.3501

[a]: x is the concentration of the compound in the reaction mixture (µM), y is the peak area ratio of the Standard over the peak area of the internal Standard (Phenacetin 50µM), a is the slope and b is the y-intercept of the linear regression.

The regression line for Testosterone substrate (R^2=0.9993) in **Table 1** demonstrates a linear range of 25- 500µM. The regression line for 16-alfa hydroxytestosterone metabolite (R^2= 0.9998) in **Table 1** demonstrates a linear range of 10- 100µM.

Table 2 illustrates the concentrations of testosterone (CYP2C11 substrate) before and after the incubation and the concentration of 16-alfa hydroxytestosterone formed after the incubation in a rat microsomal incubation assay (n=3).

Table 2. Outcomes of rat microsomal incubation assays (n=3) of 60 mins incubation using different range of Testosterone concentration levels.

Probe substrate	Amount before incubation (µM)	Amount after incubation (µM)	Metabolite	Amount after incubation (µM)
Testosterone	400	154.4205	16α-hydroxytestosterone	20.1935
	300	62.1746		18.3260
	200	7.2340		35.0633
	150	11.6789		20.3076
	100	51.9314		6.4318
	50	10.1987		4.4544
	25	0.5934		1.7575

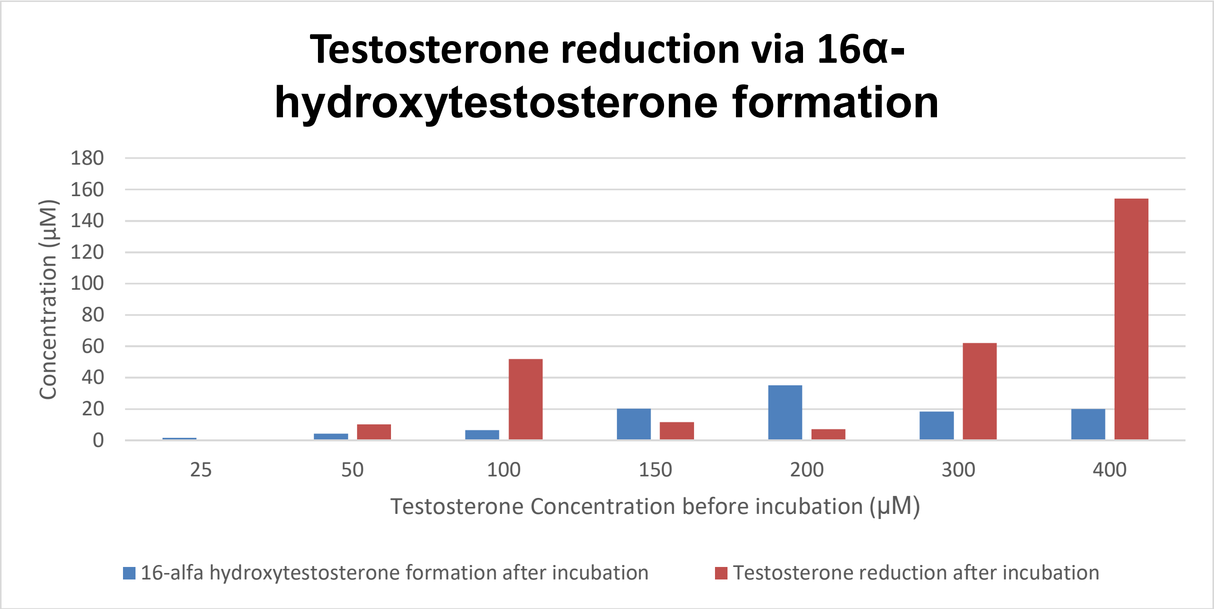

Figure 2. Bar chart shows the reduction in Testosterone and the formation of 16-alfa hydroxytestosterone after 60 mins of CYP2C11 assay incubation at different testosterone concentration levels (25, 50, 100, 150, 200, 300 and 400µM).

Table 2 and **Figure 2,** revealed that testosterone at concentration of 200µM (before incubation) metabolized to 16α-hydroxytestosterone at its maximum amount (35.0633µM) compared to other different testosterone concentrations level (400, 300, 150, 100, 50 and 25µM). Apparently, at 200µM testosterone concentration, a massive decrease in its amount was observed (from 200µM to 7.2340µM) after incubation which probably formed 16α-hydroxytestosterone at its maximum amount. Thus, in this study, Salicylic acid at different concentrations (200, 150, 100, 50 and 25µM) was incubated with testosterone at different concentrations stated (200, 150, 100, 50 and 25µM) (Yamazaki et al., 1996). Thus, testosterone at concentrations of 300 and 400µM do not necessarily need to be studied because testosterone at a concentration of 200µM formed 16α-hydroxytestosterone at its maximum amount (35.0633µM) compared to the 300 and 400µM concentrations of testosterone (18.3260 and 20.1935µM) respectively.
